# Disparities in access to care among patients with appendiceal or colorectal cancer and peritoneal metastases: A medicare insurance-based study in the United States

**DOI:** 10.3389/fonc.2022.970237

**Published:** 2022-10-31

**Authors:** Christopher T. Aquina, Zachary J. Brown, Joal D. Beane, Aslam Ejaz, Jordan M. Cloyd, Oliver S. Eng, John R.T. Monson, Samantha M. Ruff, Gyulnara G. Kasumova, Mohamed O. Adam, Samilia Obeng-Gyasi, Timothy M. Pawlik, Alex C. Kim

**Affiliations:** ^1^ Division of Surgical Oncology, Department of Surgery, The Ohio State University Wexner Medical Center, Columbus, OH, United States; ^2^ Surgical Health Outcomes Consortium (SHOC), Digestive Health and Surgery Institute, AdventHealth Orlando, Orlando, FL, United States; ^3^ Division of Surgical Oncology, Department of Surgery, University of California Irvine Medical Center, Orange, CA, United States; ^4^ Division of Surgical Oncology, Department of Surgery, University of California San Francisco Medical Center, San Francisco, CA, United States

**Keywords:** appendiceal cancer, colorectal cancer, peritoneal metastases, cytoreductive surgery (CRS) and hyperthermic intraperitoneal chemotherapy (HIPEC), healthcare disparities, access to cancer care

## Abstract

**Background:**

Prior studies attempting to identify disparities in the care of patients with appendiceal (AC) or colorectal cancer (CRC) with peritoneal metastasis (PM) are limited to single-institution, highly selected patient populations. This observational cohort study sought to identify factors associated with specialty care for Medicare beneficiaries with AC/CRC-PM.

**Materials and methods:**

Patients >65 years old in the United States diagnosed with AC/CRC and isolated PM were identified within the Medicare Standard Analytic File (2013-2017). Mixed-effects analyses assessed patient factors associated with cytoreductive surgery and hyperthermic intraperitoneal chemotherapy (CRS/HIPEC) and outpatient consultation with a peritoneal surface malignancy (PSM) surgeon, and Cox proportional-hazards analysis compared 3-year overall survival (OS) between patients receiving CRS/HIPEC versus systemic therapy alone.

**Results:**

Among 7,653 patients, only 250 (3.3%) underwent CRS/HIPEC. Among those individuals who did not undergo CRS/HIPEC (N=7,403), only 475 (6.4%) had outpatient consultation with a PSM surgeon. Patient factors independently associated with lower odds of CRS/HIPEC and PSM surgery consultation included older age, greater comorbidity burden, higher social vulnerability index, and further distance from a PSM center (p<0.05). CRS/HIPEC was independently associated with better 3-year OS compared with systemic therapy alone (HR=0.29, 95%CI=0.21-0.38).

**Conclusion:**

An exceedingly small proportion of Medicare beneficiaries with AC/CRC-PM undergo CRS/HIPEC or even have an outpatient consultation with a PSM surgeon. Significant disparities in treatment and access to care exist for patients with higher levels of social vulnerability and those that live further away from a PSM center. Future research and interventions should focus on improving access to care for these at-risk patient populations.

## Introduction

Colorectal cancer (CRC) remains the third most common cause of cancer in the United States with an incidence of 149,500 cases per year and was the third-leading cause of cancer death expected in 2021 (1). Approximately 10-15% of patients will present with peritoneal metastasis (PM) at the time of diagnosis (2). An additional 20-50% of patients will eventually develop metachronous PM (2). Although current therapies provide excellent outcomes for early-stage cancers, systemic chemotherapy is less effective for advanced stage disease, especially for PM (3). Patients with PM experience a median survival of approximately 6-8 months if untreated and approximately 16 months if treated with systemic chemotherapy (3, 4). Alternatively, cytoreductive surgery (CRS) with or without hyperthermic intraperitoneal chemotherapy (HIPEC) has been shown to be efficacious in select patients with a median survival of up to 41 months (5–7).

Due to the complexity of patients with PM, optimal disease management requires access to multiple specialists to formulate and execute a detailed treatment plan. Nevertheless, multiple barriers exist to ensuring equitable access to specialty care and oncologic outcomes. For example, previous studies have demonstrated significant gaps in access to specialty care for oncology patients across various disease sites including cervical, breast, non-small cell lung cancer, and CRC (8–11). Even after treatment, patients require frequent visits to specialists for post-treatment evaluation and cancer surveillance. For patients in vulnerable populations, which includes individuals with lower socioeconomic status, underserved ethnic minority status, and residence in rural areas, initial access to care and subsequent adherence to post-treatment care remain significant challenges (9). Given the complexity and rarity of CRS/HIPEC compared to more common oncologic operations, access to care may be even more inequitable.

Several prior studies have attempted to identify and address possible disparities related to specialty care for patients with appendiceal cancer (AC)/CRC-PM. However, these analyses were largely based on single-institution data with inherent selection bias, including pre-screening of patients prior to care, type of insurance accepted, and patients already having received care at a quaternary center (12–15). As such, a better understanding of how many patients with AC/CRC-PM are receiving specialty care and which patient factors are associated with access to referral and treatment using a non-biased approach remains crucial. In the United States, Medicare health insurance serves as universal coverage for seniors over the age of 65. Using 100% capture Medicare claims data, this study sought to identify patient factors that contributed to specialty care for patients diagnosed with AC/CRC-PM and to examine the outcome of patients following treatment.

## Materials and methods

### Data sources

#### Medicare

The Medicare 100% Inpatient Standard Analytic File (SAF) (2012-2017) was utilized to identify Medicare beneficiaries >65 years old in the United States with an initial diagnosis of AC or CRC between January 1^st^, 2013 and March 31^st^, 2017 using *International Classification of Diseases, Ninth Edition* (ICD-9) and *Tenth Edition* (ICD-10) codes. The SAF is managed by the Centers for Medicare & Medicaid Services (CMS) and includes patient-level demographics, diagnoses, procedures, and costs data from inpatient, skilled nursing facility, and hospice claims covered by Medicare Part A and outpatient and home health claims covered by Medicare Part B. The claims are linked to the Medicare Limited Data Set Denominator and Master Beneficiary Summary Files to obtain insurance status and mortality data. Medicare SAF data were available from January 1^st^, 2012 through December 31^st^, 2017. Therefore, each patient had at least one year of “look back” claims to identify the initial diagnosis of AC or CRC. The study cohort was then restricted to patients with an initial diagnosis of PM between January 1^st^, 2013 and March 31^st^, 2017 and either 60 days prior to or within 3 years following the initial diagnosis of AC or CRC. Further exclusion criteria included: 1) a diagnosis of distant metastases at other sites prior to, at the time of, or within 180 days of the initial diagnosis of PM; 2) a diagnosis of primary esophageal, gastric, small bowel, hepatopancreaticobiliary, or gynecologic cancer prior to or within 180 days following the initial diagnosis of PM; 3) non-continuous enrollment in Medicare Part A/B; 4) enrollment in a Health Maintenance Organization (HMO) health insurance plan from the date of the initial diagnosis of AC or CRC through the date of death or the end of the study period on December 31, 2017; and 5) missing county of residence for the patient. All administrative coding utilized for the study are listed in **Table 1**.

**Table 1:** Bivariate analysis of factors associated with CRS/HIPEC.

Factor	Overall study cohort(N=7,653)	No CRS/HIPEC (N=7,403) (96.7%)	CRS/HIPEC (N=250) (3.3%)	*P*
**Age** 66-69 70-79 ≥ 80	1,517 (19.8) 3,340 (43.6) 2,796 (36.5)	1,398 (18.9) 3,215 (43.4) 2,790 (37.7)	119 (47.6) 125 (50.0) 6 (2.4)	<0.001
**Sex** Male Female	3,426 (44.8) 4,227 (55.2)	3,295 (44.5) 4,108 (55.5)	131 (52.4) 119 (47.6)	0.01
**Race** White Black Other	6,737 (88.0) 558 (7.3) 358 (4.7)	6,515 (88.0) 541 (7.3) 347 (4.7)	222 (88.8) 17 (6.8) 11 (4.4)	0.93
**van Walraven Elixhauser Comorbidity Score** Median (IQR)	25 (19-33)	26 (19-33)	22 (16-30)	<0.001
**CDC Social Vulnerability Index** Median (IQR) 1^st^ quintile (least vulnerable) 2^nd^ quintile 3^rd^ quintile 4^th^ quintile 5^th^ quintile (most vulnerable)	52.4 (30.4-71.3) 1,068 (14.0) 1,607 (21.0) 1,930 (25.2) 1,866 (24.4) 1,182 (15.4)	52.6 (30.6-71.6) 1,021 (13.8) 1,532 (20.7) 1,870 (25.3) 1,821 (24.6) 1,159 (15.7)	40.6 (23.2-63.5) 47 (18.8) 75 (30.0) 60 (24.0) 45 (18.0) 23 (9.2)	<0.001 <0.001
**Distance to Nearest PSM Center** Median (IQR) < 30 miles 30-119 miles 120-239 miles ≥ 240 miles	46.6 (17.1-101.2) 2,856 (37.3) 3,346 (43.7) 1,140 (14.9) 311 (4.1)	47.0 (17.1-101.7) 2,741 (37.0) 3,245 (43.8) 1,111 (15.0) 306 (4.1)	34.2 (10.9-87.0) 115 (46.0) 101 (40.4) 29 (11.6) 5 (2.0)	0.005 0.01
**Year of Diagnosis** 2013 2014 2015 2016 2017	2,129 (27.8) 1,908 (24.9) 1,806 (23.6) 1,479 (19.3) 331 (4.3)	2,081 (28.1) 1,851 (25.0) 1,738 (23.5) 1,415 (19.1) 318 (4.3)	48 (19.2) 57 (22.8) 68 (27.2) 64 (25.6) 13 (5.2)	0.006
**Primary Cancer Site** Appendix Right colon Left colon Colon of unspecified site Rectum	678 (8.9) 3,202 (41.8) 2,109 (27.6) 925 (12.1) 739 (9.7)	522 (7.0) 3,154 (42.6) 2,073 (28.0) 922 (12.4) 732 (9.9)	156 (62.4) 48 (19.2) 36 (14.4) 3 (1.2) 7 (2.8)	<0.001
**Timing of Carcinomatosis** Synchronous Metachronous	6,027 (78.7) 1,626 (21.2)	5,813 (78.5) 1,590 (21.5)	214 (85.6) 36 (14.4)	0.007

CRS/HIPEC, cytoreductive surgery/hyperthermic intraperitoneal chemotherapy; PSM, peritoneal surface malignancy; CDC, Centers for Disease Control and Prevention.

### Outcomes

The primary outcome was treatment with CRS/HIPEC within 365 days of the date of the initial PM diagnosis. Because there are no specific ICD-9 procedure codes for CRS/HIPEC, a combination of hyperthermia and/or intraperitoneal chemotherapy procedure codes and at least one procedure code for an abdominal operation were utilized to identify CRS/HIPEC cases for inpatient claims with a discharge date prior to the implementation of ICD-10 codes on October 1^st^, 2015 as published previously in the surgical literature (16). For inpatient claims with a discharge date of October 1^st^, 2015 or later, specific ICD-10 codes for HIPEC were utilized to identify CRS/HIPEC cases (**Supplementary Table 1**).

Secondary outcomes included outpatient evaluation by a peritoneal surface malignancy (PSM) surgeon and 3-year overall survival (OS). A PSM surgeon was defined as a surgeon who performed at least one CRS/HIPEC case for a Medicare beneficiary during the study period and had a specialty taxonomy in the CMS National Plan and Provider Enumeration System (NPPES) of general surgery, surgical oncology, or colon & rectal surgery. Surgeons were matched to the NPPES using the National Provider Identifier number of the primary surgeon within the Medicare claim for the CRS/HIPEC procedure (17, 18). Three-year OS was defined as death from any cause within 3 years of the initial diagnosis date of PM. To limit heterogeneity with respect to patient fitness and treatment intent, the survival analyses only included patients who either underwent CRS/HIPEC or who received at least one cycle of systemic chemotherapy, targeted biologic therapy, or immunotherapy within 90 days of the initial diagnosis date of PM. These analyses were restricted to patients with information on CRS/HIPEC or systemic therapy within the Medicare Physician Supplier Part B/Carrier, Inpatient, and Outpatient claims.

### Covariates

Patient factors included in the study are listed in **Table 2**. “Other” race included those that were coded as other, Asian, Hispanic, or North American Native within the Limited Data Set Denominator and Master Beneficiary Summary Files. The van Walraven Elixhauser Comorbidity Score is a validated modification of the thirty Elixhauser binary comorbidity measures that uses a weighted score for each of the comorbidities to compute a single numeric score for administrative data using ICD-9/ICD-10 diagnosis codes (19, 20). The CDC Social Vulnerability Index is a county-level estimate of the population’s social vulnerability based on 15 United States census variables including socioeconomic status, household composition and disability, minority status and language, and housing type and transportation (21). Primary cancer site was categorized into appendiceal, right colon, left colon, unspecified colon site, and rectal cancer. Synchronous PM was defined as an initial PM diagnosis date within 180 days of the initial AC or CRC diagnosis date, and metachronous PM was defined as an initial PM diagnosis date 180 days or more after the initial AC or CRC diagnosis date. Distance to the nearest PSM center was estimated using the great-circle distance in miles from the county centroid of the patient’s primary residence at the time of diagnosis to the county centroid of the nearest PSM center using the Haversine formula. This information was available through the National Bureau of Economic Research (NBER) and based upon the Federal Information Processing Standard Publication (FIPS) United States county codes using 2010 U.S. census data (22). PSM centers were identified within the Medicare data and defined as hospitals that performed an average of ≥1 CRS/HIPEC cases per year for appendiceal neoplasm, CRC, gastric cancer, ovarian cancer, primary peritoneal malignancy, or PM during the study period (**Supplementary Table 1**).

**Table 2:** Mixed-effects multivariable analysis of factors associated with CRS/HIPEC.

Factor	Odds ratio (95% CI)	*P*
**Age** 66-69 70-79 ≥ 80	Reference 0.46 (0.32-0.64) 0.03 (0.01-0.06)	<0.001 <0.001
**Sex** Male Female	Reference 0.69 (0.50-0.95)	0.02
**Race** White Black Other	Reference 1.06 (0.58-1.78) 0.74 (0.33-1.55)	0.82 0.45
**van Walraven Elixhauser Comorbidity Score**	0.98 (0.96-1.00)	0.04
**CDC Social Vulnerability Index** Continuous (per 10^th^ percentile increment increase)* 1^st^ quintile (least vulnerable) 2^nd^ quartile 3^rd^ quartile 4^th^ quintile 5^th^ quintile (most vulnerable)	0.87 (0.82-0.93) Reference 1.08 (0.66-1.82) 0.77 (0.48-1.23) 0.53 (0.32-0.89) 0.45 (0.24-0.85)	<0.001 0.81 0.29 0.01 0.01
**Year of Diagnosis** 2013 2014 2015 2016 2017	Reference 1.35 (0.85-2.05) 1.49 (0.93-2.24) 1.68 (1.03-2.64) 1.44 (0.67-2.83)	0.21 0.10 0.04 0.34
**Primary Cancer Site** Appendix Right colon Left colon Colon of unspecified site Rectum	Reference 0.05 (0.04-0.07) 0.05 (0.03-0.08) 0.01 (0.003-0.03) 0.03 (0.01-0.05)	<0.001 <0.001 <0.001 <0.001
**Timing of Carcinomatosis** Synchronous Metachronous	Reference 0.85 (0.55-1.37)	0.46
**Distance to Nearest PSM Center** Continuous (per 30 mile increment increase)* < 30 miles 30-119 miles 120-239 miles ≥ 240 miles	0.97 (0.93-1.00) Reference 0.78 (0.56-1.10) 0.63 (0.36-1.04) 0.37 (0.13-0.93)	0.09 0.17 0.07 0.04

CRS/HIPEC, cytoreductive surgery/hyperthermic intraperitoneal chemotherapy; CI, confidence interval; CDC, Centers for Disease Control and Prevention; PSM, peritoneal surface malignancy.

*Separate multivariable models were used to estimate continuous variable measures for CDC Social Vulnerability Index and distance to nearest CRS/HIPEC center.

### Statistical analysis

Bivariate analyses were performed using chi-squared and Mann-Whitney U tests, and clinically appropriate factors were manually entered into multivariable analyses for the outcomes of CRS/HIPEC, outpatient evaluation by a PSM surgeon, and 3-year OS. Two-level mixed-effects multivariable analyses accounted for clustering of patients at the county level while evaluating factors associated with the outcome measures (23, 24).

For the binomial outcomes of CRS/HIPEC and outpatient evaluation by a PSM surgeon, Bayesian mixed-effects multivariable analyses were performed. Weakly informative independent normal priors were specified for the log odds ratio, variance parameters were set to 1, co-variances to 0, and the degree of belief to 0.002, and the Gibbs sampler was utilized to run Bayesian models for 13,000 Monte Carlo Markov chain iterations with a burn-in of 3,000 iterations (18, 25).

For the time-to-event outcome of 3-year OS, mixed-effects propensity-adjusted Cox proportional-hazards analysis was performed. Given the observational nature of the data and non-random assignment of treatment with CRS/HIPEC, a propensity score for each patient was estimated from the Bayesian mixed-effects multivariable analysis as the probability of undergoing CRS/HIPEC. To avoid reduction in study cohort size, the propensity score was entered as a continuous variable in the Cox proportional-hazards model as previously described (26, 27). All patients who were alive at the end of the study period, which was December 31^st^, 2017, were censored.

Bayesian mixed-effects logistic regression analyses were performed using the *MCMCglmm* package, and mixed-effects Cox proportional-hazards analyses were performed using the *coxme* package in R, version 4.1.0 (R Foundation for Statistical Computing, Vienna, Austria) (25, 28). All other analyses were performed using SAS 9.4 (SAS Institute, Cary, NC). The study was approved by the Institutional Review Board at the Ohio State University Wexner Medical Center.

## Results

### Cohort characteristics

A total of 7,653 patients met inclusion criteria. Among 22,669 patients with an initial diagnosis of AC/CRC-PM, 11,064 were excluded due to distant metastatic disease at other sites, 2,199 were excluded due to a diagnosis of another primary abdominal malignancy, 1,272 were excluded due to non-continuous enrollment in Medicare Part A/B or HMO enrollment, and 26 were excluded due to missing county of residence.

The most common primary cancer site was right-sided colon cancer (41.8%; N=3,202) followed by left-sided colon cancer (27.6%; N=2,109), unspecified colon cancer site (12.1%; N=925), rectal cancer (9.7%; N=739), and AC (8.9%; N=678). The median age of the study cohort was 76 (interquartile range [IQR]=71-83). A higher proportion of patients were female (55.2%; N=4,227), White (88.0%; N=6,737) versus Black (7.3%; N=558) or another race (4.7%; N=358), and had synchronous PM (78.7%; N=6,027) versus metachronous disease (21.2%; N=1,626). There were 83 PSM centers identified between 2013 and 2017 across the United States, and the median patient distance to the nearest PSM center was 46.6 miles (IQR=17.1-101.2).

### CRS/HIPEC

Overall, only 3.3% (N=250) of patients underwent CRS/HIPEC. When stratified by cancer type, 23.0% (N=156) of patients with AC and 1.3% (N=94) of patients with CRC underwent CRS/HIPEC. Among patients with CRC, patients with left-sided colon cancer were more likely to undergo CRS/HIPEC (1.7%; N=36) compared with right-sided colon cancer (1.5%; N=48) and rectal cancer (0.9%; N=7) (p<0.001). Patients of older age, female sex, higher comorbidity burden, higher social vulnerability, who lived further away from a PSM center, who had an earlier year of diagnosis, and who had metachronous versus synchronous PM were less likely to undergo CRS/HIPEC (all p<0.05) (**Table 1**). Patient race was not associated with CRS/HIPEC (p=0.93). Factors independently associated with lower odds of CRS/HIPEC on multivariable analysis included older age, female sex, higher comorbidity burden, higher social vulnerability, CRC compared with AC, and further distance from the patient’s residence to the nearest PSM center (all p<0.05) (**Table 2**).

### Outpatient visit with a peritoneal surface malignancy surgeon

Overall, there were 269 PSM surgeons across 83 PSM centers identified within the 2013-2017 Medicare SAF claims. Among the 7,403 patients who did not undergo CRS/HIPEC, only 6.4% (N=475) had an outpatient visit with a PSM surgeon. When stratified by cancer type, 31.2% (N=163) of patients with AC and 4.5% (N=312) of patients with CRC had an outpatient visit with a PSM surgeon. Factors independently associated with lower odds of an outpatient visit with a PSM surgeon among those who did not undergo CRS/HIPEC were older age, higher comorbidity burden, higher social vulnerability, CRC compared to AC, synchronous PM compared to metachronous PM, and greater distance from the patient’s residence to the nearest PSM center (all p<0.05) (**Table 3**).

**Table 3:** Mixed-effects multivariable analysis of factors associated with an outpatient visit with a peritoneal surface malignancy (PSM) surgeon.

Factor	Odds ratio (95% CI)	*P*
**Age** 66-69 70-79 ≥ 80	Reference 0.50 (0.39-0.65) 0.16 (0.12-0.24)	<0.001 <0.001
**Sex** Male Female	Reference 0.84 (0.68-1.07)	0.11
**Race** White Black Other	Reference 0.77 (0.48-1.19) 1.43 (0.93-2.18)	0.20 0.11
**van Walraven Elixhauser Comorbidity Score**	0.97 (0.96-0.99)	<0.001
**CDC Social Vulnerability Index** Continuous (per 10^th^ percentile increment increase)* 1^st^ quintile (least vulnerable) 2^nd^ quartile 3^rd^ quartile 4^th^ quintile 5^th^ quintile (most vulnerable)	0.90 (0.86-0.95) Reference 0.53 (0.35-0.81) 0.67 (0.43-0.96) 0.57 (0.38-0.86) 0.44 (0.29-0.73)	<0.001 0.008 0.05 0.006 <0.001
**Year of Diagnosis** 2013 2014 2015 2016 2017	Reference 1.21 (0.88-1.60) 1.09 (0.80-1.54) 1.20 (0.84-1.67) 0.92 (0.52-1.83)	0.25 0.57 0.30 0.78
**Primary Cancer Site** Appendix Right colon Left colon Colon of unspecified site Rectum	Reference 0.07 (0.5-0.09) 0.08 (0.06-0.11) 0.06 (0.04-0.10) 0.06 (0.04-0.10)	<0.001 <0.001 <0.001 <0.001
**Timing of Carcinomatosis** Metachronous Synchronous	Reference 0.73 (0.56-0.96)	0.02
**Distance to Nearest PSM Center** Continuous (per 30 mile increment increase)* < 30 miles 30-119 miles 120-239 miles ≥ 240 miles	0.97 (0.94-1.00) Reference 0.56 (0.42-0.76) 0.30 (0.20-0.48) 0.34 (0.16-0.66)	0.02 <0.001 <0.001 0.002

CRS/HIPEC, cytoreductive surgery/hyperthermic intraperitoneal chemotherapy; CI, confidence interval; CDC, Centers for Disease Control and Prevention.

*Separate multivariable models were used to estimate continuous variable measures for CDC Social Vulnerability Index and distance to nearest peritoneal surface malignancy center.

### Three-year overall survival

**Figure 1:**
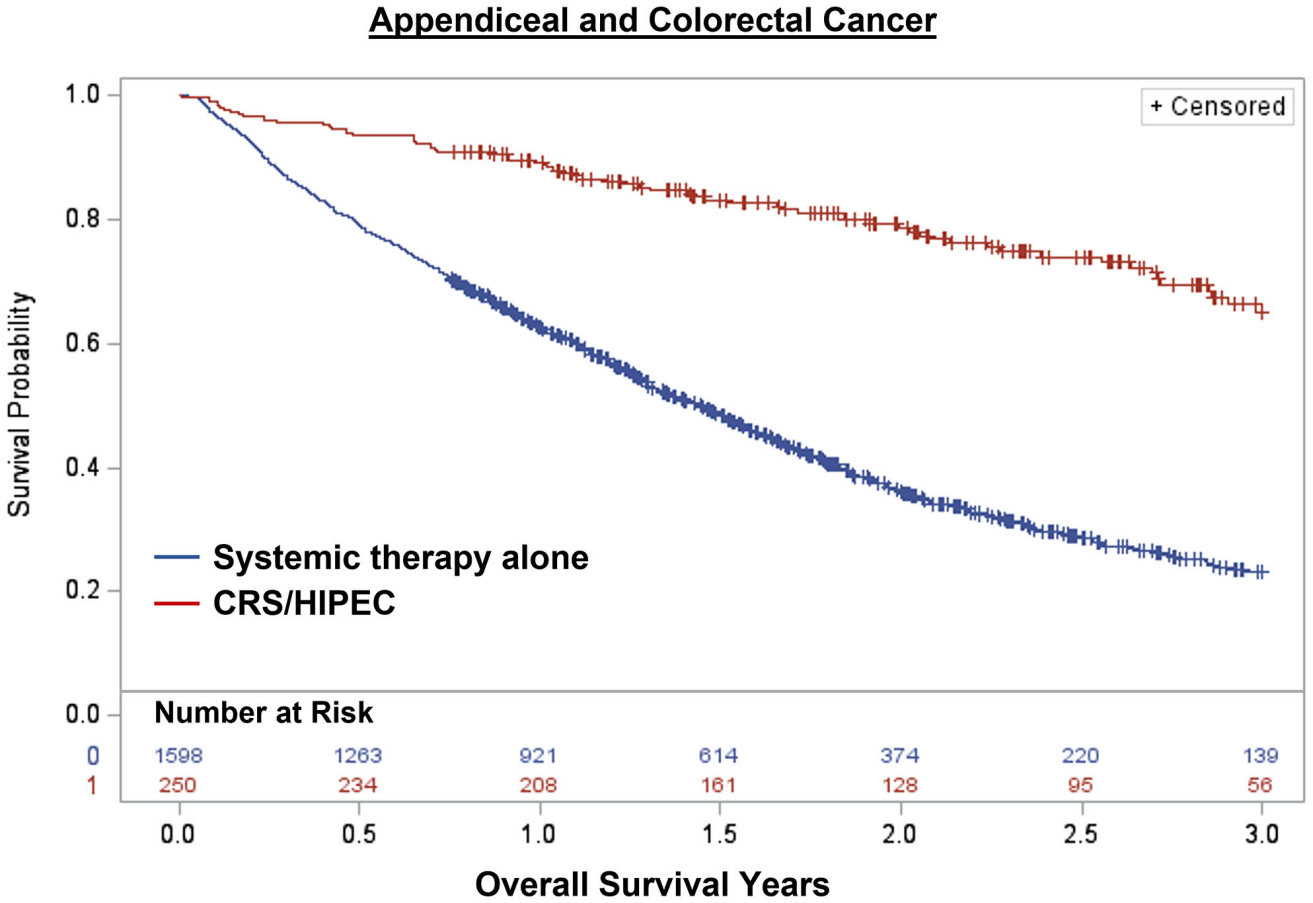
Kaplan-Meier 3-year overall survival of 1,848 patients with appendiceal or colorectal cancer and peritoneal metastasis stratified by treatment with CRS/HIPEC +/- systemic therapy or systemic therapy alone.

**Figure 2:**
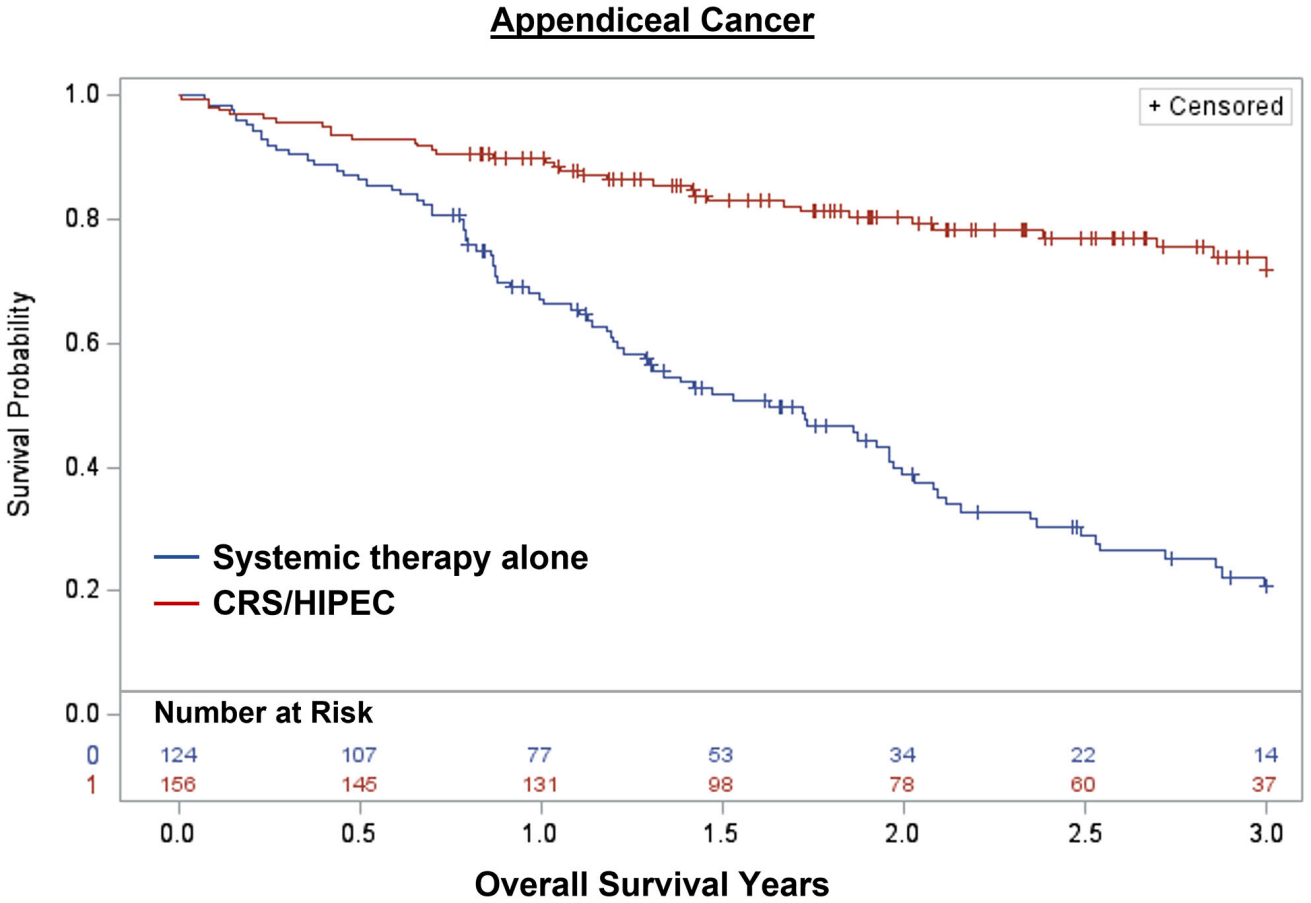
Kaplan-Meier 3-year overall survival of 280 patients with appendiceal cancer and peritoneal metastasis stratified by treatment with CRS/HIPEC +/- systemic therapy or systemic therapy alone.

**Figure 3:**
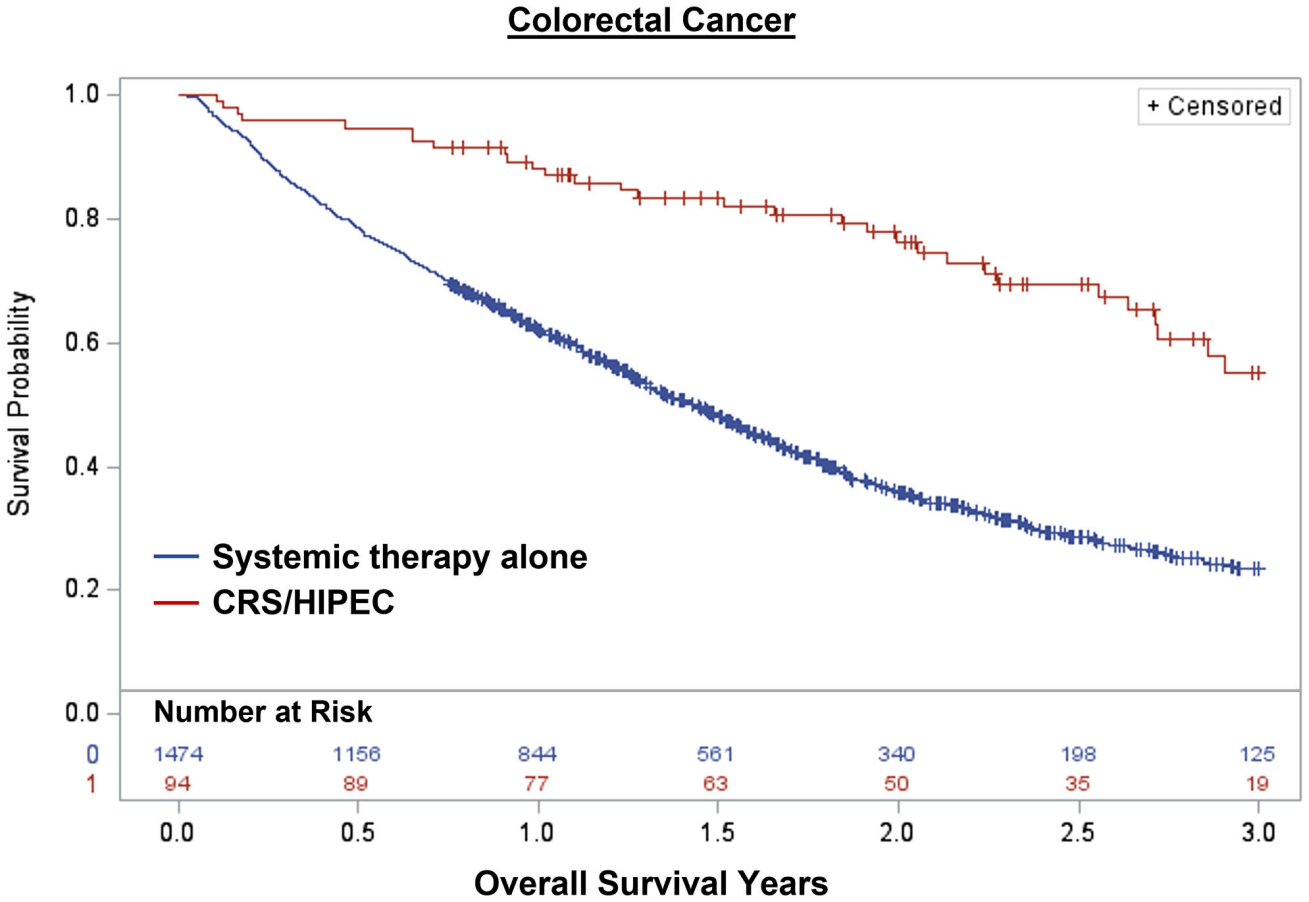
Kaplan-Meier 3-year overall survival of 1,568 patients with colorectal cancer and peritoneal metastasis stratified by treatment with CRS/HIPEC +/- systemic therapy or systemic therapy alone.

Overall, 1,848 patients were treated with CRS/HIPEC and/or systemic therapy. Comparing individuals who underwent CRS/HIPEC (13.5%; N=250) to systemic therapy alone (86.5%; N=1,598), CRS/HIPEC was associated with better 3-year OS (74.4% vs 35.1%; log-rank p<0.001). When stratified by cancer type, CRS/HIPEC was associated with better 3-year OS for both AC (78.2% vs 33.1%; log-rank p<0.001) and CRC (68.1% vs 35.3%; log-rank p<0.001) (**Figures 1**–**3**). After propensity and risk-adjustment, CRS/HIPEC was independently associated with better 3-year OS (hazard ratio [HR]=0.29, 95% confidence interval [CI]=0.21-0.38) compared to systemic therapy alone (**Table 4**).

**Table 4:** Mixed-effects propensity-adjusted Cox proportional-hazards analysis of association between CRS/HIPEC and overall survival*^,^†.

	Overall study cohort (N = 1, 848)		Appendiceal cancer (N = 280)		Colorectal cancer (N = 1, 568)	
Factor	HR (95% CI)	*P*	HR (95% CI)	*P*	HR (95% CI)	*P*
Systemic therapy alone CRS/HIPEC	Reference 0.29 (0.21-0.38)	<0.001	Reference 0.22 (0.14-0.32)	<0.001	Reference 0.35 (0.24-0.51)	<0.001

CRS/HIPEC, cytoreductive surgery/hyperthermic intraperitoneal chemotherapy; HR, hazard ratio;

CI, confidence interval.

^*^To limit study cohort heterogeneity with respect to patient fitness and treatment intent, the analysis only includes patients who underwent CRS/HIPEC or systemic therapy.

^†^Models also control for patient age, sex, race, van Walraven Elixhauser comorbidity score, CDC social vulnerability index quintile, year of diagnosis, primary cancer site, synchronous vs metachronous carcinomatosis, and distance to nearest peritoneal surface malignancy center.

## Discussion

Among Medicare beneficiaries in the United States, only 1 in 30 patients underwent CRS/HIPEC for AC/CRC-PM between 2013 and 2017. While the rate of CRS/HIPEC was higher among patients with AC-PM at 23%, the rate of CRS/HIPEC for CRC-PM was only 1.3%. Furthermore, patients with higher social vulnerability or who lived further away from a PSM center were less likely to undergo CRS/HIPEC or have outpatient consultation with a PSM surgeon. These findings highlight disparities in access to care for AC/CRC-PM patients with higher social vulnerability and/or increased travel burden. Given the recent findings from the PRODIGE-7 trial demonstrating a clear long-term survival benefit associated with CRS+/-HIPEC compared to survival data from other trials in which patients received systemic therapy alone, these findings highlight the need for future research focusing on interventions to improve access to care for this at-risk patient population (3, 4, 7).

This study is the first observational study to the authors’ knowledge to assess healthcare disparities in care for AC/CRC-PM using a national study cohort in the United States. Two prior studies investigated possible treatment-related disparities for AC/CRC-PM using the National Cancer Database (NCDB) (13, 14). However, the NCDB is not population-based as it is limited to cases diagnosed or treated at Commission-on-Cancer (CoC)-accredited institutions in the United States. In a study by Byrne et al. that included 18,055 patients with AC, White patients, non-Hispanic ethnicity, and private insurance were associated with receipt of CRS/HIPEC (13). However, the study included patients without peritoneal metastasis, and neighborhood-level socioeconomic characteristics were not assessed. In a study by Goldberg et al. that included 6,634 patients diagnosed with ovarian or CRC-PM, the rate of CRS was 18.1%, and older age, male sex, lymph node metastasis, and community hospitals versus academic centers were associated with lower odds of receiving CRS (14). Interestingly, patient median household income, education status, distance to the reporting hospital, and treatment at facilities with higher-income patient populations were not associated with receipt of CRS. However, as aforementioned, the study was limited to those treated at CoC-accredited centers.

Other prior studies were limited to single-institution data with inherent selection bias that included pre-screening of patients prior to care, types of insurance accepted, and patients already having received care at specialized centers. In a case-control study by Tabrizian et al. comparing all patients with CRC-PM who had undergone CRS/HIPEC between 1993 and 2013 (N=112) and patients who underwent either colectomy for non-metastatic colon cancer or hepatectomy for colorectal cancer liver metastasis, patients who underwent CRS/HIPEC were more likely to be White, English speaking, privately insured, have higher mean income, and travel further distances for treatment compared with the control groups (12). In a separate study by Rieser et al. that included 226 patients who underwent CRS/HIPEC for CRC-PM between 2000 to 2018 at a high-volume tertiary CRS/HIPEC center, patients with high socioeconomic status were more likely to be White, privately insured, and travel further distances for treatment compared to those with low socioeconomic status (15). Following CRS/HIPEC, patients with low socioeconomic status had worse outcomes, including longer length of stay, higher rates of 90-day readmission and 30-day mortality, and lower median OS.

Another possible disparity that was identified was related to patient sex. While the association between female sex and outpatient consultation with a PSM surgeon did not reach statistical significance (p=0.11), female sex was independently associated with lower odds of CRS/HIPEC compared to male sex. Interestingly, this difference was also observed by Byrne et al. in which male patients were 33% more likely to undergo CRS/HIPEC for appendiceal cancer compared to female patients (13). Unfortunately, the reasons for this association cannot be elucidated from the Medicare data. Possible explanations include an underlying disparity or more advanced disease at time of diagnosis. Future research is needed to better understand this association.

While the rate of CRS/HIPEC was much higher for AC-PM compared to CRC-PM (23% versus 1.3%), the reasons for the overall low rate of CRS/HIPEC in the current study are likely multifactorial. Medicare beneficiaries >65 years of age are likely to have an increased risk of postoperative complications and mortality secondary to a higher comorbidity burden and less functional reserve which may influence their perceived ability to tolerate a high-risk operation (29). However, only 5% of the 66-69 age group within the study underwent CRS/HIPEC, suggesting that the low rate of utilization also occurred across younger age groups. The availability of CRS/HIPEC was also limited, as reflected by only 83 hospitals being identified as PSM centers in the study. Of note, the median distance from the patient residence to the nearest PSM center across the study cohort was 46.6 miles. In addition, there was underutilization of referral to PSM surgeons, which is likely related to both lack of access to PSM specialists and limitations in knowledge among providers related to the postoperative outcomes and efficacy of CRS+/-HIPEC in the treatment of PSM. Furthermore, as a higher proportion of providers view CRS/HIPEC as an appropriate treatment modality for AC-PM compared to CRC-PM, a limitation in knowledge may at least partially explain the higher rates of CRS/HIPEC (23% versus 1.3%) and outpatient consultation with a PSM surgeon (31% versus 4.5%) for AC-PM compared to CRC-PM (30–32).

These suspected reasons for low rates of CRS/HIPEC and referral to PSM surgeons are supported by provider survey data. In a study by Bernaiche et al, medical oncologists and general surgeons in Virginia, Maryland, and Washington, D.C. who treated patients with gastrointestinal cancer were asked questions regarding access to centers that performed CRS/HIPEC, prior referral to PSM centers, opinions regarding efficacy of CRS/HIPEC, and knowledge with respect to postoperative outcomes following CRS/HIPEC (30). Among 116 respondents, 41% indicated that multidisciplinary tumor board discussion of patients with PM occurred ≤50% of the time, and 34% stated that PSM specialists were not easily available to their patients. For specific cancer types, CRS/HIPEC was considered an appropriate therapeutic option for AC and CRC among 75% and 50% of respondents, respectively. More than a quarter of respondents had never referred a patient to a PSM specialist in the past due to lack of access to a specialist (47%), perceived lack of efficacy of CRS/HIPEC (31%), and a belief that the morbidity and mortality of CRS/HIPEC is too high (16%). Furthermore, OS was underestimated among 48% of respondents for low-grade appendiceal mucinous neoplasm and 39% of respondents for colon cancer, and 30-day mortality at experienced PSM centers was overestimated by the majority of respondents. Similar results were observed in Ontario, Canada where only 46% of respondents were aware that CRS/HIPEC is a therapeutic option in patients with CRC-PM; in the Netherlands, 32% of providers did not view CRS/HIPEC as an accepted treatment modality for CRC-PM (31, 32).

Regardless of the possible etiologies – given the estimated annual incidence in the United States of 10,620-22,550 for CRC-PM and 600 for AC-PM – CRS/HIPEC clearly appears to be underutilized (33). There are several potential strategies to improve referral rates and access to PSM specialists for patients. Under ideal circumstances, all patients with isolated PM or PM with limited, resectable extraperitoneal metastatic disease should undergo formal multidisciplinary review with surgeons, medical oncologists, and a trained PSM surgeon. In geographic areas where there is no qualified PSM surgeon, virtual tumor board or telemedicine referral and evaluation, which has been shown to be a cost-effective modality for specialized care, are alternative options (34, 35). In light of improved perioperative outcomes and a long-term survival benefit from CRS/HIPEC in carefully selected patients, education of various stakeholders including medical providers, patients, policy makers, and payers regarding the efficacy of CRS/HIPEC for AC/CRC-PM may also lead to higher referral rates (7, 36). Furthermore, the creation of financial assistance programs with travel and lodging vouchers for disadvantaged patients with limited financial means and higher travel burden to the nearest PSM center will help reduce disparities in access to care.

While this study is the first national observational cohort study investigating factors associated with receipt of CRS/HIPEC for AC/CRC-PM, there are several limitations. Medicare SAF is susceptible to medical coding errors since it is comprised of administrative data. In addition, TNM staging is not available within the data. However, validation studies have demonstrated low false positive rates with the use of ICD-9/ICD-10 diagnosis coding algorithms to identify metastatic disease in colorectal cancer with a specificity > 90% (37, 38). Similarly tumor histology, differentiation, and disease burden as measured by the peritoneal carcinomatosis index are also not available which influences the decision on whether a patient may benefit from CRS/HIPEC. Because these factors are not available within the data, the 3-year overall survival analyses had to be limited to those who underwent CRS/HIPEC or systemic therapy alone to reduce heterogeneity among patients. Furthermore, because there are no specific codes for CRS, it was not possible to identify patients who underwent CRS without HIPEC which can also lead to long-term survival (7). However, CRS is rarely performed without HIPEC for AC/CRC-PM (39). Finally, the study cohort was necessarily restricted to patients > 65 years old with Medicare insurance. The rates of CRS/HIPEC are likely higher in younger patient populations who are healthier and have better functional status.

## Conclusion

An exceedingly small proportion of Medicare beneficiaries with AC/CRC-PM undergo CRS/HIPEC or even have an outpatient consultation with a PSM surgeon. Significant disparities in treatment and access to care were evident for patients with higher levels of social vulnerability and who live further away from a PSM center. Future research should focus on interventions to improve referral rates to PSM centers and appropriate access to care for these at-risk patient populations.

## Data availability statement

The data analyzed in this study is subject to the following licenses/restrictions: The Medicare Standard Analytical claims data contain patient-level health information and are considered identifiable files. Therefore, access to these data requires a data use agreement and institutional review board approval. Requests to access these datasets should be directed to https://www.cms.gov/Research-Statistics-Data-and-Systems/Files-for-Order/LimitedDataSets/StandardAnalyticalFiles.

## Ethics statement

The studies involving human participants were reviewed and approved by Institutional Review Board at the Ohio State University Wexner Medical Center. Written informed consent for participation was not required for this study in accordance with the national legislation and the institutional requirements.

## Author contributions

CA and AK contributed to the conception and design of the work. CA, ZB, JB, AE, JC, OE, JM, SR, GK, MA, SO-G, TP, and AK contributed to acquisition, analysis, and/or interpretation of data for the work. CA and AK contributed to drafting of the work. ZB, JB, AE, JC, OE, JM, SR, GK, MA, SO-G, TP, and AK contributed to revising the work for important intellectual content. CA, ZB, JB, AE, JC, OE, JM, SR, GK, MA, SO-G, TP, and AK provided approval for publication of the work and agreement to be accountable for all aspects of the work in ensuring that questions related to the accuracy or integrity of any part of the work are appropriately investigated and resolved. All authors contributed to the article and approved the submitted version.

## Acknowledgment

Data within this article were previously presented as an oral presentation at the Society of Surgical Oncology 2022 Advanced Cancer Therapies meeting on March 12th, 2022, in Dallas, TX.

## Conflict of interest

Author AE was employed by Natera, Inc. and Delcath, Inc.

The remaining authors declare that the research was conducted in the absence of any commercial or financial relationships that could be construed as a potential conflict of interest.

## Publisher’s note

All claims expressed in this article are solely those of the authors and do not necessarily represent those of their affiliated organizations, or those of the publisher, the editors and the reviewers. Any product that may be evaluated in this article, or claim that may be made by its manufacturer, is not guaranteed or endorsed by the publisher.
