# Supplementary material for: Disparities in access to care among patients with appendiceal or colorectal cancer and peritoneal metastases: A medicare insurance-based study in the United States
Source: Front Oncol. 2022 Oct 31;12:970237. doi: 10.3389/fonc.2022.970237 (PMC9659914; doi:10.3389/fonc.2022.970237)
Supplement: Supplementary file 1 [file Table_1.docx]

**Supplemental Table 1**: Administrative Coding Utilized for the Study

| **Cancer Diagnoses** |  |
| --- | --- |
| Appendiceal cancer | 153.5  C18.1 |
| Colorectal cancer | Right colon cancer  153.0-153.1, 153.4, 153.6  C18.0, C18.2-C18.4  Left colon cancer  153.2-153.3, 153.7, 154.0  C18.5-C18.7, C19  Colon of unspecified site  153.8-153.9  C18.8-C18.9  Rectal cancer  154.1  C20 |
| Peritoneal metastasis | 158.8-158.9, 197.6  C48.1-C48.8, C78.6 |
| Distant metastasis | Liver metastasis  155.2, 197.7  C22.9, C78.7  Lung/intrathoracic metastasis  197.0-197.3  C78.00-C78.39  Brain metastasis  198.3  C79.31-C79.32  Adrenal metastasis  198.7  C79.70-C79.72 |
| Other primary abdominal cancer | Esophageal cancer  150.0-151.0  C15.3-C16.0  Gastric cancer  151.1-151.9  C16.1-C16.9  Small bowel cancer  152.0-152.9  C17.0-C17.9  Hepatopancreaticobiliary cancer  155.0-155.1, 156.0-157.9  C22.0-C22.8, C23-C25.9  Gynecologic cancer  179-184.9  C51.0-C58 |
| **Operations** |  |
| Abdominal operations | Laparotomy  54.11-54.19, 54.59  Laparoscopy  54.21, 54.51  Peritonectomy  54.4  Diaphragm excision  34.81  Colectomy  17.31-17.39, 45.71-45.83  Proctectomy  48.5-48.63  Appendectomy  47.01-47.19  Bowel anastomosis  45.90-45.95  Gastrectomy  43.5-43.99  Splenectomy  41.5  Cholecystectomy  51.22-51.23  Hepatectomy  50.22, 50.3  Small bowel resection  45.61-45.62  Oophorectomy  65.31-65.64  Hysterectomy  68.31-68.9  Cystectomy  57.71-57.79, 68.8  Ileostomy  46.01, 46.20-46.23  Colostomy  46.03, 46.10-46.13, 48.5-48.59, 48.62 |
| HIPEC | 3E0M305, 3E0M705, 3E0M30Y |
| Hyperthermia for treatment of cancer | 99.85 |
| Implantation of chemotherapy | 00.10 |
| Injection of locally-acting therapeutic  substance into peritoneal cavity | 54.97 |
| Injection/infusion of chemotherapy | 99.25 |
| Possible HIPEC | 1. Hyperthermia for treatment of cancer   *OR*   1. Implantation of chemotherapy   *OR*   1. Injection of locally-acting therapeutic substance into peritoneal cavity *AND* injection/infusion of chemotherapy *AND* date of injection of locally-acting therapeutic substance into peritoneal cavity = date of injection/infusion of chemotherapy |
| CRS/HIPEC | 1. HIPEC   *OR*   1. Abdominal operation *AND* possible HIPEC *AND* date of abdominal operation = date of possible HIPEC |
| **Systemic therapy** |  |
| Diagnosis codes | V58.11-V58.12, V66.2, V67.2  Z51.11-Z51.12 |
| Procedure codes | 99.25, 99.28  3E03005, 3E0300M, 3E03305, 3E0330M, 3E04005, 3E0400M, 3E04305, 3E0430M |
| HCPCS codes | 5-Fluorouracil  J9190  Leucovorin  J0640-J0642  Oxaliplatin  J9263  Irinotecan  J9205-J9206  Capecitabine  J8520-J8521  Bevacizumab  J9035  Cetuximab  J9055  Panitumumab  J9303  Nivolumab  J9299  Ipilimumab  J9228  Pembrolizumab  J9271  Ramucirumab  J9308  Ziv-aflibercept  J9400 |
| National drug code | 5-Fluorouracil  00187-3204, 00187-5200, 00378-4791, 00378-8078, 16110-0812,  16729-0276, 25021-0215, 28105-0421, 50090-0426, 50742-0423,  50742-0481, 50742-0482, 50742-0483, 51672-0406, 51672-0411,  51862-0362, 63323-0117, 66530-0249, 68001-0266, 68083-0269,  68083-0270, 68083-0292, 68083-0293, 70700-0186, 70700-0187,  70700-0188, 70700-0189  Leucovorin  00054-4496, 00054-4497, 00054-4498, 00054-4499, 00054-8496,  00143-9368, 00143-9552, 00143-9553, 00143-9554, 00143-9555,  00143-9558, 00555-0484, 00555-0485, 00703-5140, 00703-5145,  00781-3201, 00904-6703, 16714-0890, 16714-0915, 25021-0813,  25021-0814, 25021-0815, 25021-0816, 25021-0828, 42806-0133,  42806-0134, 42806-0358, 42806-0359, 43598-0771, 43598-0773,  50742-0181, 50742-0182, 50742-0183, 50742-0184, 50742-0464,  50742-0494, 50742-0495, 51079-0581, 51079-0582, 60687-0227,  63323-0631, 63323-0710, 63323-0711, 64661-0650, 64661-0711,  67457-0528, 67457-0529, 67457-0530, 68001-0416, 68001-0417,  68001-0418, 68083-0278, 68083-0279, 68152-0112, 68152-0114,  69315-0184, 69315-0185, 69315-0186, 69315-0187, 70121-0109,  70121-0157, 71205-0908, 71288-0104, 71288-0105, 72266-0120,  72266-0121, 72893-0004, 72893-0006, 72893-0009, 72893-0013,  72893-0014  Oxaliplatin  00703-3985, 00703-3986, 00781-3315, 00781-3317, 00781-9315,  00781-9317, 00955-1725, 00955-1727, 00955-1731, 00955-1733,  16714-0727, 16714-0728, 16729-0332, 25021-0233, 43066-0014,  43066-0018, 45963-0611, 50742-0405, 50742-0406, 55150-0331,  55150-0332, 60505-0613, 61703-0363, 63323-0750, 67184-0050,  67184-0051, 67457-0442, 67457-0469, 68001-0468, 68083-0170,  68083-0171, 68083-0176, 68083-0177, 68083-0314, 69097-0274,  69097-0353, 70860-0201, 71288-0101, 71288-0149, 72266-0125,  72266-0126, 72266-0161, 72266-0162, 72603-0101, 72603-0301,  79672-0825, 79672-0826  Irinotecan  00009-7529, 00143-9583, 00143-9701, 00143-9702, 15054-0004,  16714-0027, 16714-0131, 25021-0230, 45963-0614, 50742-0401,  50742-0402, 55150-0352, 55150-0353, 55150-0354, 59923-0702,  59923-0714, 59923-0715, 59923-0716, 60505-0612, 61703-0349,  63323-0193, 67184-0051, 68001-0480, 68083-0381, 68083-0382,  70700-0169, 70700-0170, 72485-0211, 72485-0212, 72485-0213  Capecitabine  00004-1100, 00004-1101, 00054-0271, 00054-0272, 00093-7473,  00093-7474, 00378-2511, 00378-2512, 16714-0467, 16714-0468,  16729-0072, 16729-0073, 50268-0154, 51079-0510, 51407-0095,  51407-0096, 55111-0496, 55111-0497, 59651-0204, 59651-0205,  59923-0721, 59923-0722, 60687-0149, 62756-0238, 62756-0239,  64980-0276, 64980-0277, 65162-0843, 65162-0844, 67877-0458,  67877-0459, 68001-0487, 68001-0488, 69097-0948, 69097-0949,  69539-0019, 69539-0020, 70756-0815, 70756-0816, 72205-0006,  72205-0007, 72485-0204, 72485-0205, 72606-0554, 72606-0555  Bevacizumab  00069-0315, 00069-0342, 50242-0060, 50242-0061, 55513-0206,  55513-0207  Cetuximab  66733-0948, 66733-0958  Panitumumab  55513-0954, 55513-0956  Nivolumab  00003-3734, 00003-3772, 00003-3774  Ipilimumab  00003-2327,00003-2328  Pembrolizumab  00006-3026  Entrectinib  50242-0091, 50242-0094  Ramucirumab  00002-7669, 00002-7678  Regorafenib  50419-0171  Encorafenib  70255-0025  Larotrectinib  50419-0390, 50419-0391, 50419-0392, 71777-0390, 71777-0391,  71777-0392  Ziv-aflibercept  00024-5840, 00024-5841, 61755-0005  Trifluridine-tipiracil  50383-0955, 61314-0044, 64842-0102 |
| Revenue center code | 0331-0332, 0335 |
| BETOS code | 01D |
| **Surgeon Taxonomy** |  |
| General surgery | 208600000X |
| Surgical oncology | 2086X0206X |
| Colon and rectal surgery | 208C00000X |

HIPEC= hyperthermic intraperitoneal chemotherapy; HCPCS=Healthcare Common Procedure Coding System; BETOS=Berenson-Eggers Type of Service
